# Extensive Inter-Cyst DNA Methylation Variation in Autosomal Dominant Polycystic Kidney Disease Revealed by Genome Scale Sequencing

**DOI:** 10.3389/fgene.2020.00348

**Published:** 2020-04-15

**Authors:** Sarah A. Bowden, Peter A. Stockwell, Euan J. Rodger, Matthew F. Parry, Michael R. Eccles, Cherie Stayner, Aniruddha Chatterjee

**Affiliations:** ^1^Department of Pathology, Dunedin School of Medicine, University of Otago, Dunedin, New Zealand; ^2^Maurice Wilkins Centre for Molecular Biodiscovery, Auckland, New Zealand; ^3^Department of Mathematics and Statistics, University of Otago, Dunedin, New Zealand

**Keywords:** polycystic kidney, DNA methylation, autosomal dominant polycystic kidney disease, reduced representation bisulfite sequencing, epigenetics

## Abstract

Autosomal dominant polycystic kidney disease (ADPKD) is a heritable disease characterized by bilateral renal enlargement due to the growth of cysts throughout the kidneys. Inheritance of a disease-causing mutation is required to develop ADPKD, which results in end-stage kidney disease and is associated with a high morbidity. The pathology underlying cyst formation is not well understood. To address this, we have previously shown the global methylome is altered in ADPKD tissue, suggesting a role of DNA methylation in disease-state renal tissue. As cysts are believed to arise independently, we hypothesize that DNA methylation changes vary accordingly. Here we further investigate the role of DNA methylation within independent cysts to characterize key intra-individual changes. We demonstrate that fragments within CpG islands and gene bodies harbor the greatest amount of variation across the ADPKD kidney, while intergenic fragments are comparatively stable. A proportion of variably methylated genes were also differentially methylated in ADPKD tissue. Our data provide evidence that individual molecular mechanisms are operating in the development of each cyst.

## Introduction

Autosomal dominant polycystic kidney disease (ADPKD) is characterized by the bilateral accumulation of fluid-filled cysts within the kidneys. Ultimately, disease progression results in kidney enlargement, leading to end-stage kidney disease typically by the sixth decade (Braun, 2009). While ADPKD is the most common heritable renal disease in humans, affecting an estimated 1–5 in 10,000 patients (Solazzo et al., 2018), treatment options for this disease are limited as there is still little understanding about the underlying cause of cystogenesis (Tan et al., 2011; Reed-Gitomer, 2014).

The presence of a disease-causing mutation is required to develop ADPKD, however, a causal relationship between these mutations and the process of cystogenesis is not yet clearly elucidated. PKD-causing mutations are primarily found within the *PKD1* (85%) and *PKD2* (15%) genes. However, in atypical presentations of ADPKD, disease-causing mutations have also been reported in *GANAB* (Porath et al., 2016) and *DNAJB11* (Cornec-Le Gall et al., 2018). It is generally accepted that each ADPKD cyst arises by means of focal proliferation (Reed-Gitomer, 2014), where a single cell will acquire altered growth characteristics, rapidly proliferate and form a distinct cyst.

DNA methylation is required for the regulation of gene expression (Herman and Baylin, 2003; Yang et al., 2014), and therefore tissue-specific methylation must be conserved in order for the normal functioning of genes (Tang et al., 2016). Studies on DNA methylation have thus traditionally investigated differential methylation between two groups. However, in recent years, the study of variation in the methylome has become of increasing interest.

The identification of CpG sites which do not necessarily show differential methylation between populations, but instead exhibit DNA methylation variation across a population has been suggested as a response to the cellular microenvironment (Hansen et al., 2011) or chemical exposure (Phillips et al., 2019). Furthermore, DNA methylation variation has been observed amongst individuals with autoimmune diseases (Paul et al., 2016; Webster et al., 2018) and intra-individually in chronic lymphocytic leukemia samples (Landau et al., 2014).

Cysts are thought to arise independently, either through a second “hit” or somatic mutation in a disease-causing gene (Kim and Park, 2016), or by other mechanisms that modify gene expression, such as epigenetic changes (Woo, 2016). Previous work on DNA methylation in ADPKD has been conducted at bulk-tissue level (Woo et al., 2014; Bowden et al., 2018). As bulk tissue samples will likely contain cells from multiple cysts, as well as stromal and parenchymal cells, the methylation profile will reflect the average of all cell types and therefore may not accurately represent the profile of epithelial cells that line the cyst. To address this, we have performed reduced representation bisulfite sequencing (RRBS), targeting single cyst walls to sequence discreet populations of enriched clonal cystic epithelia and to validate the data collected from bulk tissue analyses. To further describe the role of DNA methylation in cystogenesis, we decided to investigate the methylomes of individual ADPKD cysts from a single ADPKD patient in order to validate the whole tissue data. We hypothesize that each cyst arises independently, and therefore carries its own methylome, which would demonstrate that individual molecular mechanisms are operating in each cyst.

Initial investigation led us to discover discrete sites of variation across a single patient kidney, which we have characterized. The regions in which we have observed this variation suggest that each ADPKD cyst is responding independently to cellular stress.

## Methods

### Cyst Dissection From a Single ADPKD Kidney

**FIGURE 1:**
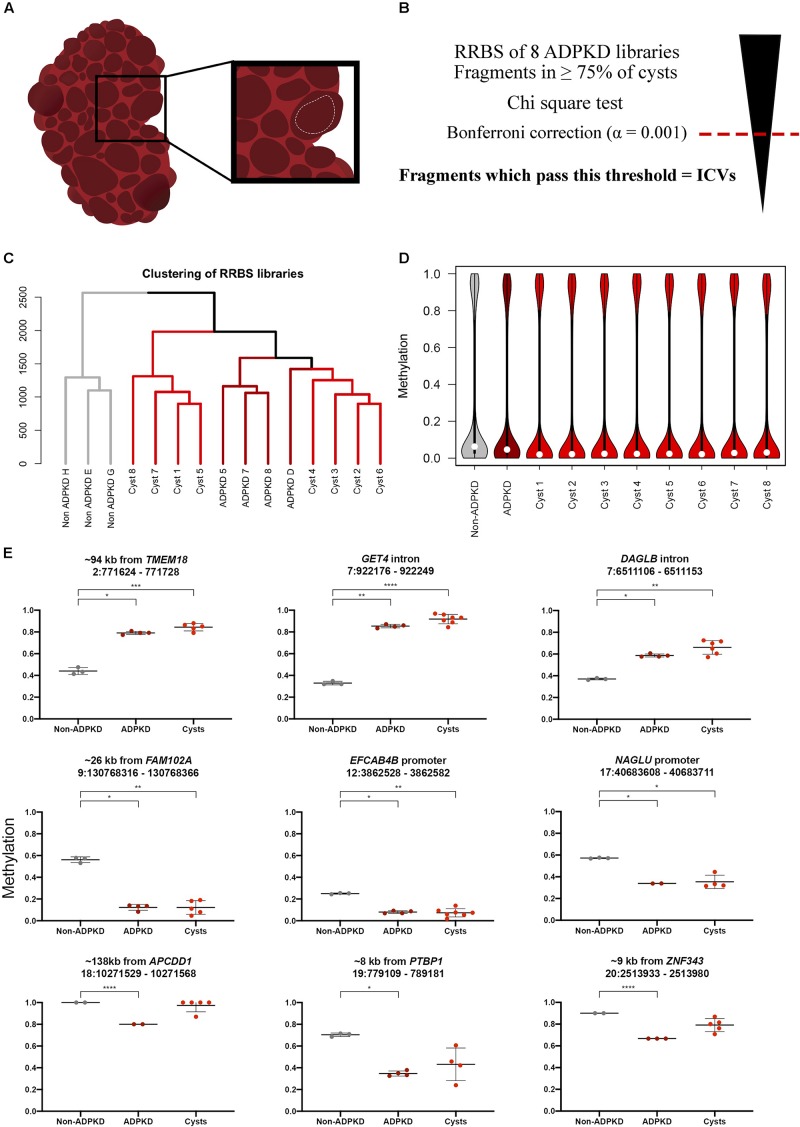
Individual ADPKD cysts show the same methylation pattern as whole tissue ADPKD samples globally and at specific fragments. **(A)** Fluid-filled cysts were visually identified throughout the patient kidney following nephrectomy. Sections of the cyst wall were cut out with surgical scissors (white dotted line), creating sections of tissue approximately 1 cm^2^, with the intention to avoid neighboring tissue. **(B)** ICVs were classified by first performing a Chi-square test on the analyzed fragments. The Bonferroni correction (α = 0.001) was used to select the most variable fragments from this analysis; fragments with *p*-values below this threshold were classed as ICVs. **(C)** Clustering of the non-ADPKD (*n* = 3), ADPKD whole tissue (*n* = 4) and ADPKD cyst (*n* = 8) RRBS libraries over all common analyzed fragments (*n* = 39,708) reveals non-ADPKD clusters separately to ADPKD tissue. Cyst samples are clustered into two distinct branches. **(D)** Distribution of global methylation in each RRBS library. White dot denotes median methylation. Analysis of common analyzed fragments across all samples shows that cysts are globally hypomethylated compared to non-ADPKD tissue. Notably, cysts are more hypomethylated than the whole tissue ADPKD counterpart. **(E)** Nine of the previously identified DMFs had coverage in ≥50% of the ADPKD cysts (three with <50% coverage in Supplementary Figure S1), and six of these showed a statistically significant difference from non-ADPKD tissue as determined by ANOVA. Data from ADPKD tissue previously published (Bowden et al., 2018), FDR-adjusted *p*-values: * < 0.05, ** < 0.01, *** < 0.001, **** < 0.0001.

Three non-ADPKD cortical kidney tissue samples were provided from the Christchurch Cancer Society Tissue Bank, as previously described (Bowden et al., 2018). These samples were collected from the renal cortex during nephrectomy for renal cell carcinoma, with histological testing to confirm the absence of malignant tissue. Eight single cyst samples were macrodissected from a single ADPKD kidney (male, 60 years old, *PKD1* mutation c.3744_3745dup (het), NM_001009944.2). Following nephrectomy, the ADPKD kidney was stored in neutral buffered formalin for 7 days before we were able to collect it. The cyst samples were macrodissected from various sites throughout the kidney by cutting sections of the cyst wall, approximately 1 cm^2^ in size, with surgical scissors (Figure 1A). An effort was made to ensure these samples were from a single cyst; this means samples were selected from the largest intact cysts available. Tissue was washed with 70% EtOH to remove non-cyst cells (i.e., blood). The sections of cyst were then submerged in fresh 70% EtOH and stored at 4°C prior to DNA isolation. The collection and use of renal tissue was approved by the Otago University Human Ethics Committee H15/110.

### Generating Cyst Methylomes Using RRBS

DNA methylation was assessed using RRBS. RRBS library generation of the non-ADPKD renal tissue has previously been described [data accessible on the NCBI GEO database, accession GSE110057 (Bowden et al., 2018)] and is based on previously published methodologies (Chatterjee et al., 2012a, 2013, 2017a). The generation of the cyst libraries followed the same protocol as the whole renal tissue, with modifications made for the limited tissue availability and formalin fixation. Briefly, genomic DNA was extracted from the cyst samples using the QIAamp DNA FFPE Mini Kit (Qiagen), excluding the paraffin melting steps, with an extended 16-h Proteinase K treatment at 56°C. An overnight MspI digest was performed, followed by end-repair, A-tailing and ligation of methylated adapters to the resulting fragments (Illumina). Bisulfite conversion was performed using the MethylCode DNA Methylation Kit (Invitrogen).

Libraries were amplified with PCR (15–20 cycles) and run through 3% NuSieve GTG Agarose gels (Lonza) to select for fragments 150–330 bp post-adapter ligation. Libraries were quantified with the Qubit fluorometer (Life Technologies) and assessed for quality on the 2100 Bioanalyzer using the High Sensitivity DNA chip (Agilent Technologies) according to manufacturer’s instructions. Libraries with high amounts of adapter contamination (identified as peaks just under 150 bp on the Bioanalyzer trace) were re-size selected with AMPure XP Bead Purification (New England Biolabs) before repeating Qubit and Bioanalyzer steps. RRBS libraries were sequenced in an Illumina HiSeq 2500 to produce single-ended 100 bp reads. Base calling was performed with Illumina Real Time Analyzer software.

### Processing of RRBS Methylome Sequencing Data

Quality of the sequenced reads was assessed using the FastQC^1^ program. Adapter sequences and methylated CpG bases at the 3′ end of the reads that were added during the end-repair step of the library preparation were removed using our in-house *cleanadaptors* program as part of the DMAP package (Chatterjee et al., 2012b). The sequenced reads were aligned to the human genome GRCh37 with Bismark v0.6.4 alignment tools (Krueger and Andrews, 2011). We performed stringent mapping and allowed for only one mismatch in the seed (the first 28 bp of the read).

The bioinformatic software program DMAP (Stockwell et al., 2014) was used to generate MspI fragment-based methylomes and perform analysis on the sequenced reads of each sample. A non-ADPKD reference library was made by merging the three non-ADPKD tissue libraries with the program SamTools (Li et al., 2009). Fragments were only included in analyses if they satisfied our coverage criteria (contained at least two CpGs and were covered by 10 or more sequenced reads). We have demonstrated the utility of this analysis approach previously (Chatterjee et al., 2017b).

### Analysis of the Global Methylome

Direct comparison between the previous whole tissue work and the cyst libraries was performed by generating a list of fragments common in all 10 RRBS libraries (non-ADPKD renal tissue, ADPKD whole tissue, eight ADPKD cysts), in order to prevent any bias due to sequencing coverage. Previously identified differentially methylated fragments (DMFs) were assessed in the eight ADPKD cysts by performing an *F*-test analysis of variance (ANOVA) which allowed us to assess the significance in difference between methylation in the two groups at these specific fragments. Clustering analysis was performed using unsupervised hierarchical clustering in R, using the “complete” method.

Cyst-specific DMFs were identified in each cyst by performing Fisher’s exact test between each cyst library and the non-ADPKD cortical tissue reference library we generated. DMFs were selected using the Bonferroni correction for multiple testing (*p* = α/*n*; α = 0.001, cut off values in Supplementary Table S1), and an absolute difference in methylation greater than or equal to 0.25 (i.e., 25%) between ADPKD and non-ADPKD tissue. Allosomes were excluded from the analyses between multiple individuals due to sex differences between the two groups. This is different to the ANOVA test above as this is identifying changes specific to the single cyst rather than within between two groups of whole tissue which contain multiple cysts.

### Identification of Variable Inter-Cyst Fragments

To identify inter-cyst variation, a Chi-square test was performed with *n* – 2 degrees of freedom (where *n* is the number of individual cysts investigated). This resulted in the analysis of 45,954 fragments (referred to as “total analyzed fragments”). To control for multiple testing, the Bonferroni correction was applied with a stringent significance value of 0.001; the adjusted cut off *p*-value was 2.18 × 10^–8^. Fragments with *p*-values below this threshold were deemed inter-cyst variants (ICVs) (Figure 1B). An overlap between ICVs and DMFs common in ≥50% of the cysts identified variably DMFs. The cut off was 50% of cysts to account for some of the variably methylated fragments having the same methylation value as the non-ADPKD library.

### Genomic Characterization of Fragments

The *identgeneloc* program of the DMAP package was used to label fragments based on the distance to the transcription start site (TSS) of a gene. Intragenic fragments were subclassified as promoters (between 5 kb upstream and 1 kb downstream of the TSS), and those within the gene body (>1 kb downstream of the TSS) were labeled as intron, exon, and intron/exon boundary fragments by *identgeneloc*. All fragments not within this defined intragenic region were classed as intergenic fragments. *Identgeneloc* also allows for fragments to be classified based on their CpG feature as annotated by SeqMonk^2^; that is, whether they are in the core, shore or shelf of a CpG island (CGI).

Gene ontology was performed using Metascape (Zhou et al., 2019). Gene lists (Supplementary Tables S3–S7) were analyzed alongside a list of background genes (*n* = 17,917), which was generated from a list of all sequenced fragments in the nine RRBS libraries. A list of genes (*n* = 2,415) associated with intragenic ICVs (*n* = 4,204) was analyzed to characterize pathways associated with ICVs, as intragenic fragments were more likely to have consequential effects on gene transcription.

Genes were ranked with a variation score, to indicate the susceptibility of the gene to DNA methylation variation. This was calculated by dividing the number of intragenic ICVs associated with the gene, divided by the total number of intragenic fragments associated with the gene in the analysis. Genes were required to contain at least three ICVs in order to be included in the analysis as this conferred a reasonable amount of certainty about the amount of variation in the gene. Fragments with a variation score of 1.0 had total variation in all sequenced fragments in the analysis.

### Statistics

To identify inter-cyst variation, a Chi-square test was performed with *n* – 2 degrees of freedom (where *n* is the number of cysts investigated). To control for multiple testing, the Bonferroni correction with a stringent significance value of 0.001 was applied to each analysis to determine the *p*-value threshold. The statistical significance for other analyses (such as the proportion of ICVs in the chromosome and genomic features) were calculated with a Chi-square test with Yate’s correction, where *p* < 0.001 was the minimum value for significance. To plot the distribution of *p*-values, QQ-plots were generated using R (Supplementary Figure S6).

## Results

### Collection of ADPKD Single Cysts

Cysts were visually confirmed to be throughout the kidney upon dissection. As the kidney had been cut up for formalin fixation many had drained, but some still contained cyst fluid. The larger of the cysts were collected in order to extract as much DNA for analysis as possible. These had thicker membranes and were more distinct from the stroma. As the kidney was partially dissected when we had access to it, we could not determine the spatial organization of the cysts in the kidney.

### ADPKD Cysts Resemble ADPKD Tissue at the Global Methylome Level

We carried out genome-wide bisulfite sequencing on eight single kidney cyst samples from an ADPKD patient using RRBS. We used this data alongside previously described whole renal tissue RRBS data from non-ADPKD and ADPKD kidneys (Bowden et al., 2018). We first wanted to compare global methylation data from the eight ADPKD cysts to ADPKD whole tissue samples and non-ADPKD samples which we had previously generated. The non-ADPKD samples clearly clustered separately from the cysts and ADPKD tissue samples (Figure 1C). Next, we identified the global change in median methylation in each cyst in comparison to non-ADPKD and ADPKD whole tissue samples using only the common analyzed fragments, where it was observed that all eight ADPKD cysts were hypomethylated by 3–4% in comparison to our non-ADPKD reference genome (Figure 1D and Supplementary Table S2). This is consistent with our whole tissue data which showed a 2% reduction in median methylation in ADPKD compared to non-ADPKD. When compared to non-ADPKD using the same common analyzed fragments, the whole tissue ADPKD samples were only hypomethylated by 1.6%.

We then sought to validate the previously identified DMFs in each cyst to confirm that these changes are universal in each ADPKD cyst. There was coverage in ≥50% of the cysts within nine of the whole tissue DMFs, with statistically significant differences between non-ADPKD tissue and ADPKD cysts in six DMFs (Figure 1E).

### ADPKD Cysts Are Differentially Methylated Across Developmental Pathways

**FIGURE 2:**
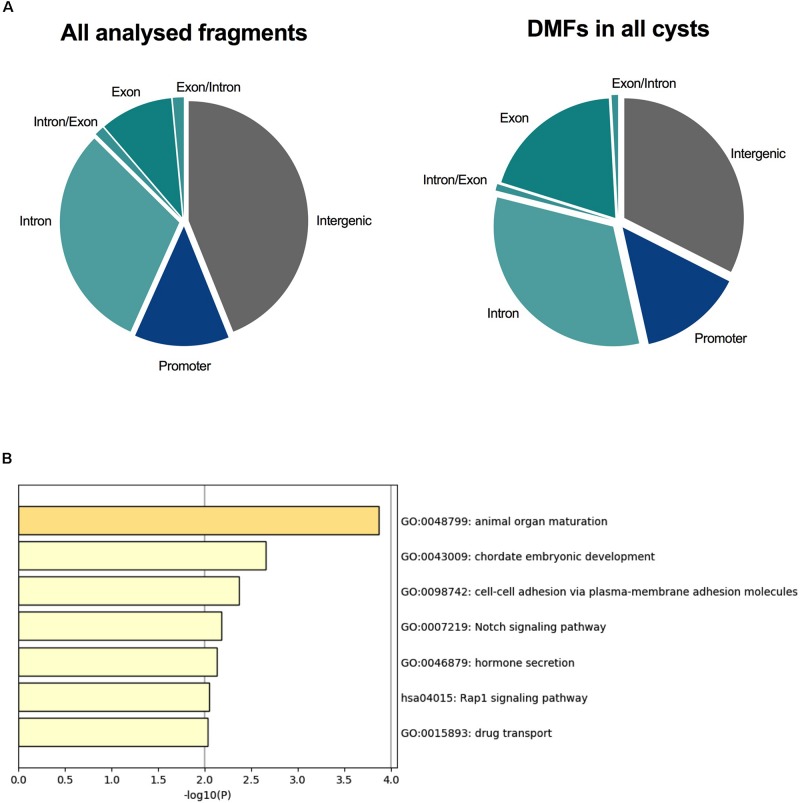
All eight ADPKD cysts were differentially methylated across PKD-related pathways. **(A)** The proportion of fragments associated with each genomic element in the analysis. Intergenic fragments (both upstream and downstream of protein-coding genes) are gray. Promoter fragments are blue, and fragments within the gene body are shades of teal. Ninety-six DMFs were common in all eight cysts, with 54% of these occurring within the gene body. **(B)** Gene ontology identified common enriched pathways associated with these fragments. Animal organ maturation was the most significantly enriched, followed by embryonic development. Interestingly, cell-cell adhesion and Notch signaling, both associated with ADPKD, were also enriched in these differentially methylated fragments.

Differential methylation has largely been the focus of DNA methylation analyses, since such changes in the promoters of genes may confer stable changes in gene expression (Jones, 2012; Chatterjee et al., 2018). For this reason, we decided to identify DMFs within each ADPKD cyst to determine whether key changes occurred within each individual cyst. An analysis was performed between each ADPKD cyst and the non-ADPKD methylome using Fisher’s exact test. The number of DMFs identified in cysts (Supplementary Table S1) ranged from 474 (in Cyst 4) to 6,087 (in Cyst 1). Of these fragments, 96 common DMFs, which all demonstrated the same direction of methylation change, were identified in all eight cysts. The common DMFs were slightly enriched within the gene body of protein-coding genes (54%; Figure 2A) and were enriched for pathways associated with animal organ maturation as well as cell-cell adhesion and Notch signaling (Figure 2B and Supplementary Table S9).

Of our previously identified whole tissue DMFs (Bowden et al., 2018), the *TMEM18*- and *GET4*-associated fragments were also differentially methylated in some of the single cysts according to the stricter criteria (Supplementary Figure S2).

### Inter-Cyst DNA Methylation Variation Is Enriched in Intragenic Regions

As illustrated by comparison to our DMF data, there was a significant amount of variability across the eight cysts. To quantify this further, we selected fragments present in at least six of the eight cysts (to account for lower methylome coverage in some cysts), which resulted in 45,954 total analyzed fragments. Fragments displaying significant inter-cyst variation were identified by applying a Chi-square goodness of fit test, followed by the Bonferroni adjustment for multiple test correction at a significance level of 0.001 (adjusted *p*-value cut off = 2.18 × 10^–8^). This analysis identified 6,727 fragments (14.6% of the total analyzed fragments) which were termed “inter-cyst variants” (ICVs).

**FIGURE 3:**
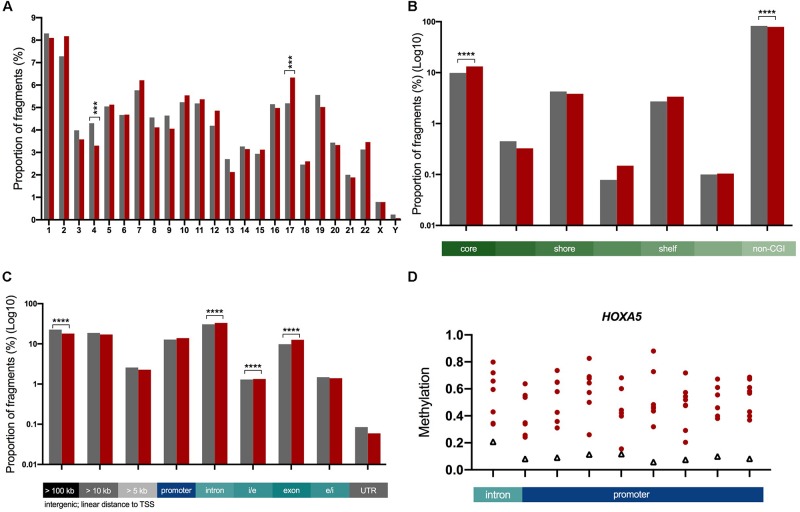
Characterization of ICVs in the genome. The proportion of ICVs in each genomic element (red, *n* = 6,727) were compared to the proportion of all fragments (gray, *n* = 45,954). Statistical significance assessed with a Chi-square test with Yate’s correction, ***p < 0.001, ****p < 0.0001. Bar graphs: gray = total analyzed fragments, red = ICVs. **(A)** A significant change in the proportion of ICVs as determined by a Chi-square test with Yate’s correction test is seen in chromosomes 4 and 17. **(B)** The proportion of ICVs within and across boundaries of the CGI features, such as CGI shore, show that CGIs predominantly harbor more variation. This is shown in log10 scale as there are significantly more fragments outside CGIs than within. **(C)** ICVs were underrepresented in intergenic fragments (gray) and overrepresented in intragenic fragments (blue, teal). Exons harbor the greatest amount of enrichment (28% increase in the proportion of fragments). Scaled to log10 to illustrate changes in all genomic elements. **(D)** *HOXA5* is one of 18 highly variable genes across ADPKD cysts. Nine highly variable fragments spread across 1,633 bp. All 18 in Supplementary Figure S3. Red circles = individual cysts, black triangles = non-ADPKD reference methylome.

Next we characterized the ICVs based on their chromosomal and genomic location. We found that the proportion of ICVs in two chromosomes (4 and 17) was significantly different to the proportion of total analyzed fragments by ≥12% (*p* = 0.001, Figure 3A). When ICVs were characterized by relation to CGIs, ICVs were shown to be more strongly enriched in fragments within the core of the CGI (Figure 3B). The proportion of ICVs increased in intragenic fragments and conversely decreased in intergenic fragments (Figure 3C).

Genes were ranked with a variation score, which was generated based on how many fragments associated with each gene were variably methylated, in order to determine the genes with the most variable methylation states. A total of 18 genes (Figure 3D and Supplementary Figure S3) had a variation score of 1.0 (at least three fragments associated with the gene, all classed as ICVs), and were functionally enriched for the gene ontology processes sulfur compound metabolic processes and GTPase regulation (Supplementary Figure S4 and Supplementary Table S8).

When the null hypotheses are true, the distribution of *p*-values is uniform on the interval (0, 1). The histogram of actual *p*-values (Supplementary Figure S5) shows a pronounced skew toward values near zero. The QQ-plot (Supplementary Figure S6) displays the same information: observed quantiles lie below the quantiles of the uniform distribution (indicated by the 45-degree line).

### Genes Involved in Developmental Pathways Are Enriched for DNA Methylation Variation

In this analysis there were 4,204 intragenic ICV fragments, associated with 2,415 unique genes. We investigated the functional pathways of this group of genes, as methylation at these fragments is historically considered more likely to impact the gene function than methylation within intergenic fragments (Klose and Bird, 2006).

Gene ontology analysis through Metascape revealed the intragenic ICV-associated genes were enriched for a number of developmental and morphogenesis pathways (Supplementary Figure S7 and Supplementary Table S10; intergenic ICV-associated gene ontology data in Supplementary Table S11).

### Variably Methylated Fragments Are Also Differentially Methylated in ADPKD

As there were differences in the number of ICVs identified in each cyst, we aimed to investigate if there was an overlap between DMFs and ICVs, i.e., were fragments which showed variable methylation measurably different to the non-ADPKD tissue.

**FIGURE 4:**
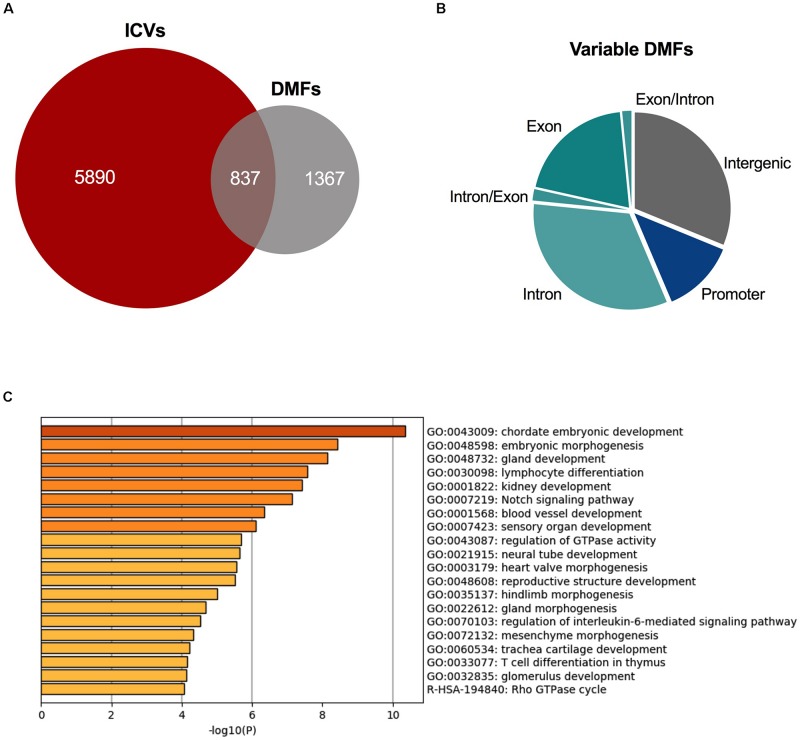
Variably methylated fragments are also differentially methylated in ADPKD. **(A)** There were 837 DMFs across the ADPKD kidney that also demonstrated significant inter-cyst variation. **(B)** These variable DMFs are largely present in the intragenic fragments. Comparison to ICVs and DMFs in Supplementary Figure S8. **(C)** These variable DMFs are enriched for many development and differentiation pathways, including embryonic development and morphogenesis, and kidney development. Top 20 terms identified by metascape.org displayed here (full list in Supplementary Table S12).

A total of 2,204 fragments were differentially methylated in at least 50% of the cysts. 837 of these DMFs shared an overlap with the 6,727 ICVs (Figure 4A), occurring in 586 unique genes. These fragments which overlapped with DMFs and ICVs could be said to be variable DMFs. Variable DMFs were predominantly located within the gene body of protein-coding genes (Figure 4B), which were functionally enriched for development pathways (Figure 4C).

We previously identified 13 DMFs in ADPKD tissue and have now identified *TMEM18-* and *GET4-* associated differential methylation also occurs individually in single cysts. These DMFs did not demonstrate variable methylation.

## Discussion

The role of DNA methylation in ADPKD is an emerging field of study and following our recent identification of site-specific DNA methylation changes in cystic renal tissue, we sought to investigate if these changes are seen consistently between individual cysts in a single patient. We have identified that in addition to whole tissue DMFs being present in most single cysts, there are regions of heterogeneity in the DNA methylation of cysts across a single individual ADPKD patient.

The cysts in this analysis were large, and visually confirmed to be cysts before microdissection took place. Due to the limited availability of tissue, no IHC or protein analysis was possible to confirm the phenotypic nature of the samples.

Using our criteria for assessing variation, 14.6% of the fragments in our analysis were identified as ICVs. ICVs represent areas of the genome with marked methylation variability across ≥75% of the cysts, suggesting possible instability of these particular sites or elements in ADPKD. ICVs are overrepresented across intragenic fragments and are conversely underrepresented in the intergenic fragments. The enrichment of ICVs appears to overlap with transcriptional activity of the genome, as methylated CpGs associated with gene promoters (and sometimes gene bodies) have been shown to influence the transcriptional activity of genes (Herman and Baylin, 2003; Yang et al., 2014). Potentially, the variation of DNA methylation in the cysts could be associated with transcriptional activity of the genome.

DNA methylation variation was enriched in the core of CGIs. CGIs are regions with an increased GC density and thus higher CpG content, and are considered to be transcriptional regulators, much like gene promoters (Deaton and Bird, 2011; Jones, 2012). Therefore, the variation changes we see in the CGI cores likely act in the same manner as intragenic variation.

Interestingly, previous data generated from an intra-individual analysis of chronic lymphocytic leukemia identified a similar pattern of variation (described as proportion of discordant reads) across the genome (Landau et al., 2014). In this study, DNA methylation variation at the promoter was associated with reduced mean gene expression, but increased gene expression variation.

The intragenic ICVs suggest that the pathways with the most significant variation in methylation tend to target developmental pathways. This may be indicative of stochastic variation in these cysts, where cysts are in a transitional state between embryonic and developed tissue. Developmental changes are a feature of ADPKD cells, with embryonic transcription factors such as *PAX2* being persistently expressed in cystic epithelia (Stayner et al., 2006), and polarization defects such as the mis-localization of membrane bound proteins such as EGFR and the Na^+^K^+^ATPase, more closely resembling a fetal cellular distribution (Wilson et al., 1991).

There were 18 genes which showed total variation in our analysis (Supplementary Figure S3), representing the most contiguous regions of variable methylation in the cyst population. These variable genes are functionally associated with sulfur metabolism pathways and GTPase function, although an association between these data and pathogenesis is not yet clear. Several papers have attributed DNA methylation variation to stochastic acquisition (Landau et al., 2014; Franzen et al., 2017), which may better explain the distribution of variance observed in a single ADPKD kidney.

One possible explanation for the observed DNA methylation variation is DNA damage repair (DDR) occurring within the genome. When DNA suffers damage such as double stranded breaks (DSBs), not only must the DNA undergo repair, but also the associated epigenetic marks must also be replaced (Benayoun et al., 2015). By inducing DSBs in stem cells, Cuozzo et al. (2007) demonstrated that corresponding *de novo* methylation occurs in cells with reduced DNA homologous recombination mechanisms. Another group demonstrated that DSBs initiated the onset of CGIs promoter methylation, aberrantly silencing gene expression (O’Hagan et al., 2008).

In ciliopathies, the relationship between cellular stress and the role of DDR pathways is becoming increasingly apparent (Johnson and Collis, 2016; Zhang et al., 2019). Largely identified in nephronophthisis, mutations within DDR genes such as *ZNF423, CEP164, NME3*, and *AATF* are sufficient to cause disease via ciliary dysfunction (Chaki et al., 2012; Hoff et al., 2018; Jain et al., 2019). Additionally, *FAN1* inactivation triggers chronic kidney disease in zebrafish via a similar mechanism (Zhou et al., 2012).

Our single cyst data clustered into two separate groups based on an analysis of common analyzed fragments with unsupervised hierarchical clustering. One reason for this may be that the cysts originated from different regions of the nephron (i.e., proximal and distal tubule) which may each have unique methylome profiles. Further analysis of single cyst variation was assessed by Principal Component Analysis (PCA) (data not shown) which could not sufficiently explain the total variation seen across the ADPKD kidney by the subgrouping of cysts.

We have previously identified DMFs across bulk sections of ADPKD tissue, which contained multiple cysts. The whole tissue DMFs were (mostly) differentially methylated in the ADPKD cyst group. We did not identify these fragments as ICVs in the cyst population, which indicates that the differential methylation of ADPKD tissue is a distinct biological phenomenon to the variation of DNA methylation across an ADPKD kidney. This also supports our previous finding that dysregulation of methylation at these sites in the genome is required for ADPKD development (and thus variable methylation cannot occur here).

There are several lines of enquiry needed to follow up this study. Analyses of single cysts from additional patients would help determine the ubiquity of the variable regions we have already identified or show if they are patient specific. Single cell analyses of normal kidney would also provide suitable controls with which to validate these intriguing findings.

Cell-specific analysis of renal tubules may also help elucidate whether variation and subgrouping seen in the cyst samples is due to differences in the cell of origin or if these changes are the result of cysts following two different patterns of methylation changes during cystogenesis.

We have found that the DMF data previously generated from whole ADPKD tissue is largely reflected in the methylomes of individual ADPKD cysts. In addition to this, we found variable methylation across cysts arising from a single patient, supporting the hypothesis that there are also individual molecular mechanisms operating in the development of each cyst.

## Data Availability Statement

The datasets generated for this study can be found in the NCBI Gene Expression Omnibus https://www.ncbi.nlm.nih.gov/geo/query/acc.cgi?acc=GSE142664. The additional datasets analyzed in this study can be found in the NCBI Gene Expression Omnibus https://www.ncbi.nlm.nih.gov/geo/query/acc.cgi?acc=GSE110057.

## Ethics Statement

The studies involving human participants were reviewed and approved by the Otago University Human Ethics Committee H15/110. The patients/participants provided their written informed consent to participate in this study.

## Author Contributions

SB carried out the experimental work, performed the data analyses and wrote the first draft of the manuscript. ER and PS contributed to the data analysis. MP contributed to the statistical analysis. ME provided the critical revision and contributed in the design of the project and supervised the progression. CS conceived the idea and obtained the funding for this project. AC and CS designed and supervised the study, wrote the manuscript together with SB. AC contributed in the analysis framework of the study together with SB. All authors contributed to manuscript revision, read and approved the submitted version.

## Conflict of Interest

The authors declare that the research was conducted in the absence of any commercial or financial relationships that could be construed as a potential conflict of interest.

## Footnotes

1 http://www.bioinformatics.babraham.ac.uk/projects/fastqc/

2 https://www.bioinformatics.babraham.ac.uk/projects/seqmonk/
