# Supplementary material for: Extensive Inter-Cyst DNA Methylation Variation in Autosomal Dominant Polycystic Kidney Disease Revealed by Genome Scale Sequencing
Source: Front Genet. 2020 Apr 15;11:348. doi: 10.3389/fgene.2020.00348 (PMC7174623; doi:10.3389/fgene.2020.00348)
Supplement: Supplementary file 1 [file Data_Sheet_1.zip › New folder (2)/Table S1-2 Figures S1-8.docx]

Supplementary Material

**Supplementary Table S1: Bonferroni corrected *p* values for each cyst, and the resulting number of DMFs.** Bonferroni adjustment was made with the threshold α = 0.001, DMFs are required to have a difference in methylation between ADPKD and non-ADPKD greater than 25%. Data excludes fragments aligned to the allosomes.

| **Cyst** | **Autosomal fragments in Fisher’s Exact Test analysis** | **Bonferroni corrected**  ***p* value** | **DMFs** | **DMFs as % of all fragments** |
| --- | --- | --- | --- | --- |
| **1** | 151,878 | 6.58 x 10^-9^ | 6,084 | 4.01 |
| **2** | 159,992 | 6.25 x 10^-9^ | 3,953 | 2.47 |
| **3** | 153,690 | 6.50 x 10^-9^ | 3,926 | 2.55 |
| **4** | 29,307 | 3.41 x 10^-8^ | 474 | 1.62 |
| **5** | 81,870 | 1.22 x 10^-8^ | 2,241 | 2.74 |
| **6** | 124,500 | 8.03 x 10^-9^ | 3,572 | 2.87 |
| **7** | 54,741 | 1.83 x 10^-8^ | 1,868 | 3.41 |
| **8** | 68,485 | 1.46 x 10^-8^ | 2,268 | 3.31 |

**Supplementary Table S2: Median methylation values of each RRBS library.** Median methylomes calculated from common analysed fragments (*n* = 39,708) as shown in Figure 1B.

| **Sample** | **Median methylation** | **Difference from non-ADPKD sample** |
| --- | --- | --- |
| **Non-ADPKD** | 0.0627 | - |
| **ADPKD** | 0.0464 | 0.0163 |
| **Cyst 1** | 0.0196 | 0.0431 |
| **Cyst 2** | 0.0214 | 0.0413 |
| **Cyst 3** | 0.0235 | 0.0392 |
| **Cyst 4** | 0.0231 | 0.0396 |
| **Cyst 5** | 0.0235 | 0.0391 |
| **Cyst 6** | 0.0213 | 0.0415 |
| **Cyst 7** | 0.0278 | 0.0349 |
| **Cyst 8** | 0.0303 | 0.0323 |


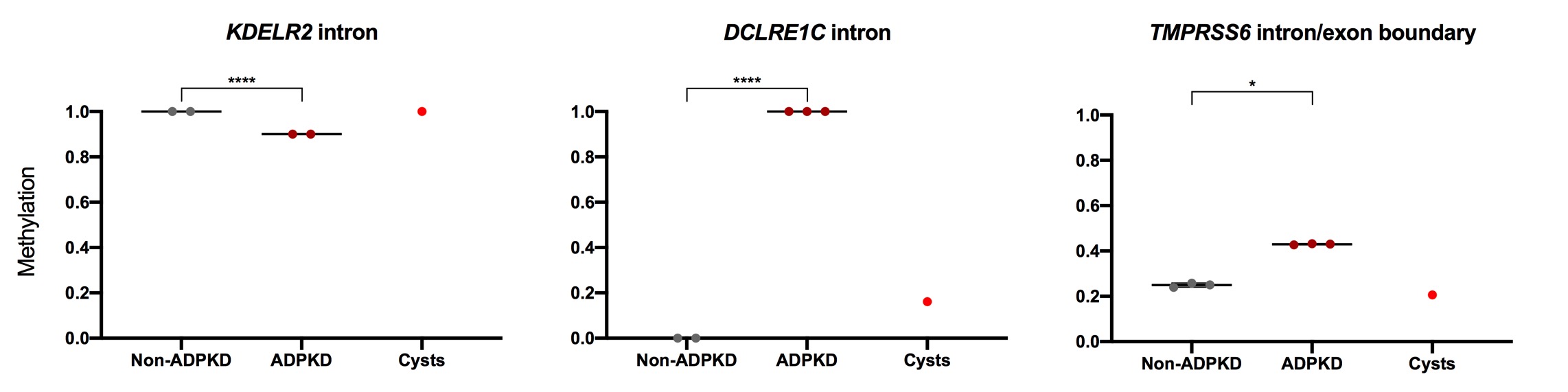


**Supplementary Figure S1: Previously identified DMFs in less than 50% of the cysts.** There was insufficient coverage at the previously identified DMFs associated with these three genes (< 50%) of the cysts. *P* values could not be ascertained with ANOVA as there was only coverage in one cyst.

**
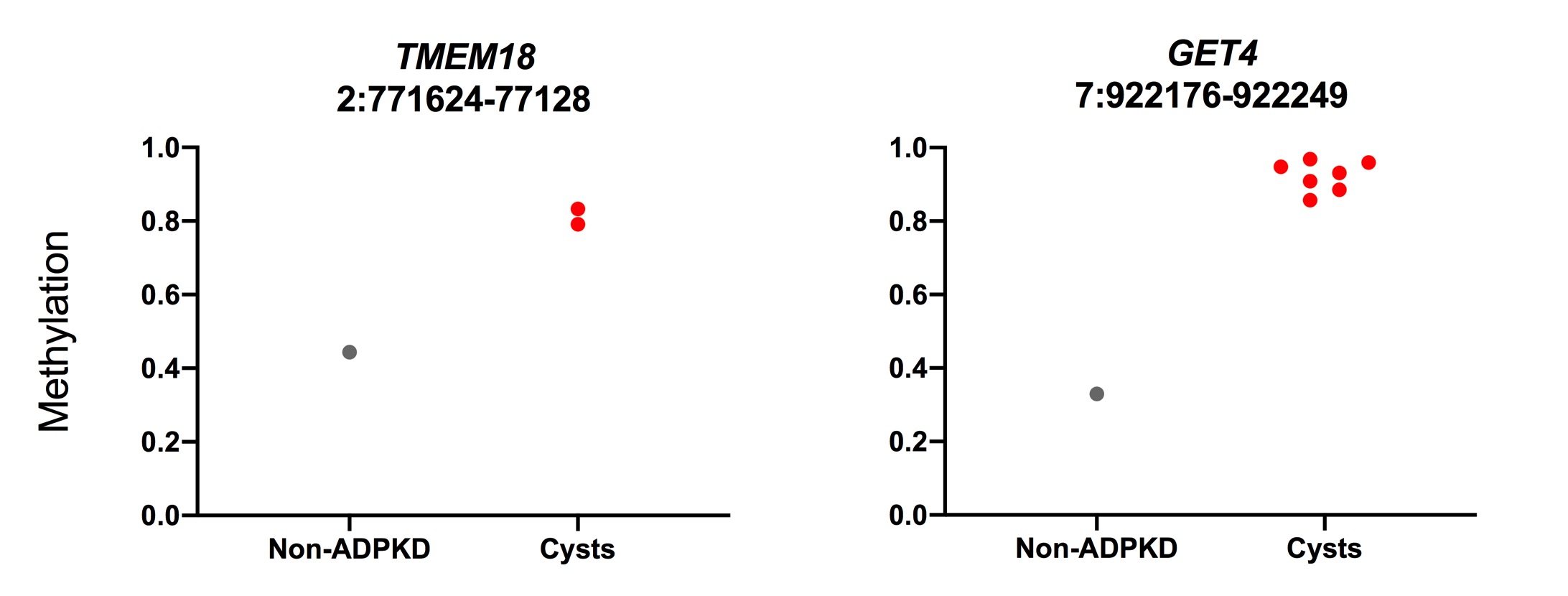
**

**Supplementary Figure S2: Previously identified DMFs associated with the genes *TMEM18* and *GET4* are differentially methylated in individual ADPKD cysts according to more stringent criteria.** Fragments were classed as DMFs in individual cysts by performing Fishers Exact Test between the non-ADPKD reference methylome and each cyst. DMFs had a difference in methylation ≥ 25% and *p* values were calculated through the Bonferroni correction (α = 0.001, Supplementary Table S1).

**
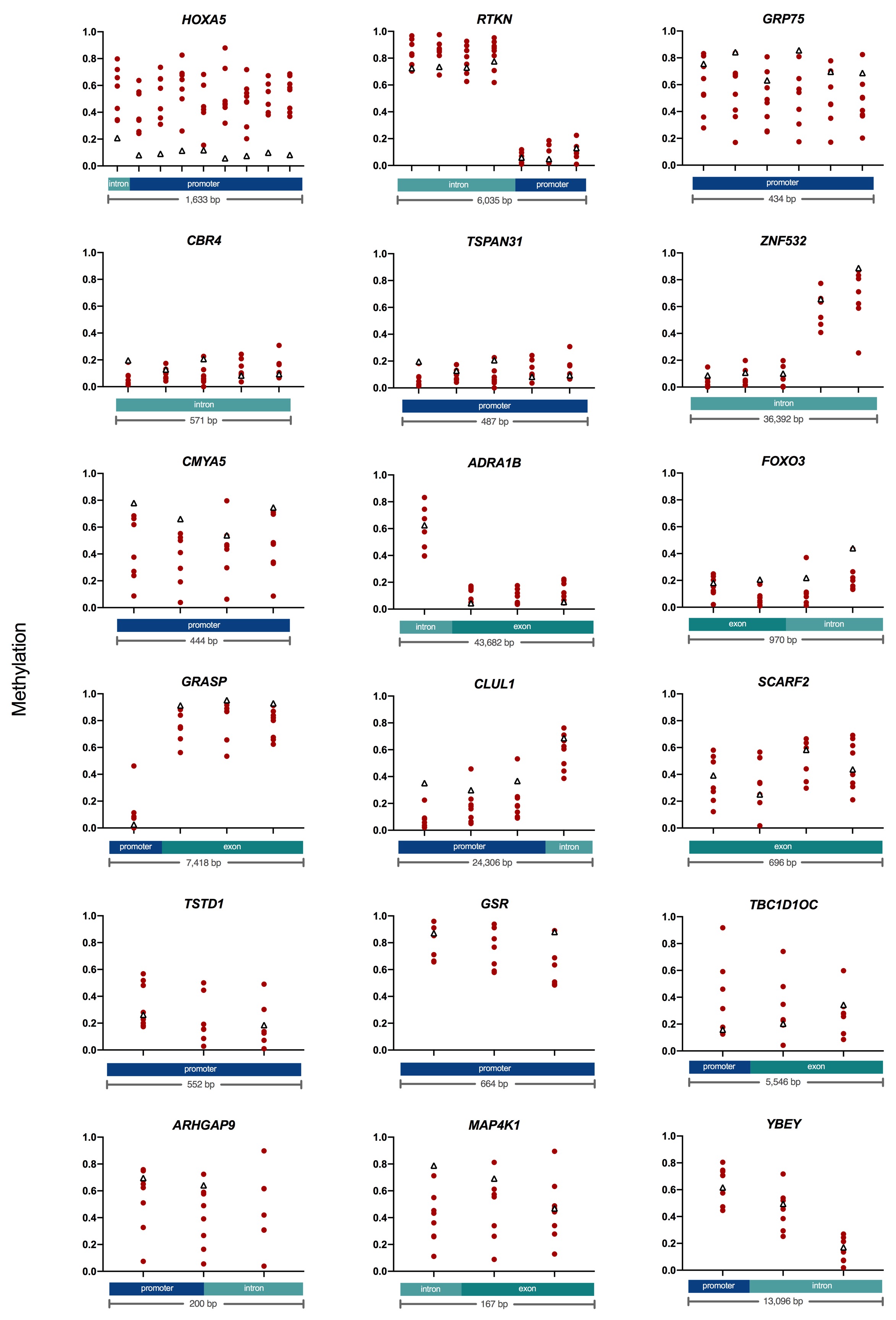
**

**Supplementary Figure S3: Top variable genes across ADPKD cysts.** There were 18 genes which had a variation score of 1.0 (total variation across all analysed fragments in the analysis, coverage in at least three fragments) within intragenic fragments only. The non-ADPKD reference methylome is represented by black triangles, red circles are individual cysts. Methylation values are superimposed at each fragment.

**
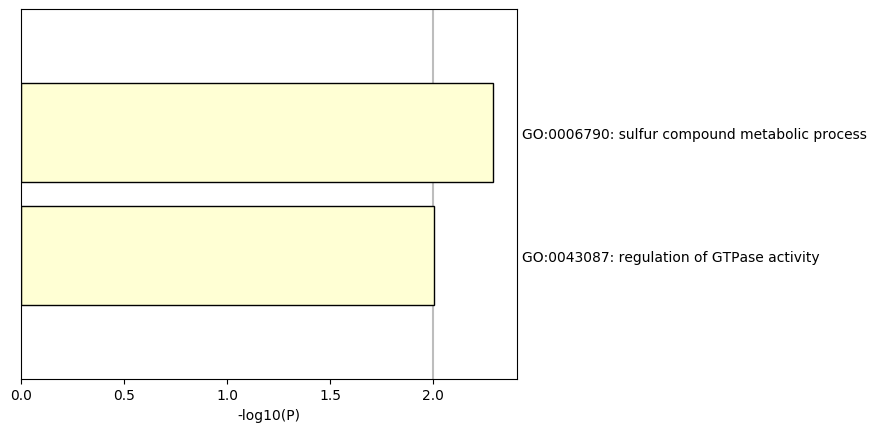
**

**Supplementary Figure S4: Gene ontology enrichment of fragments associated with top variable ADPKD cysts.** There were 18 variably methylated genes identified across the ADPKD cysts, which were enriched for two biological processes. GO gene data in Supplementary Table S8.**
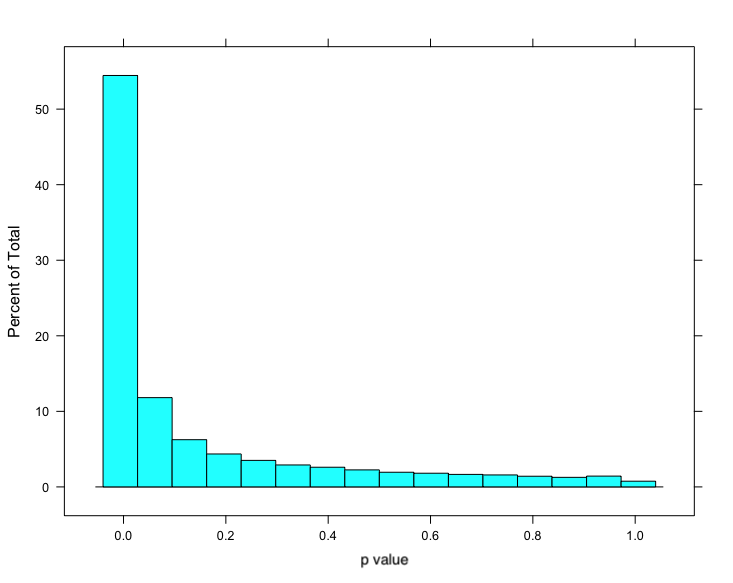
Supplementary Figure S5: Distribution of *p* values across ICV analysis.** *p* values for fragments in the chi square analysis (*n* = 45,954) show a pronounced skew towards values near zero.

**
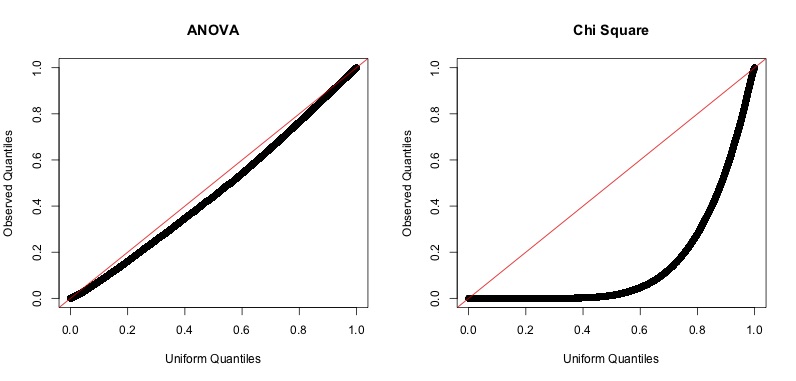

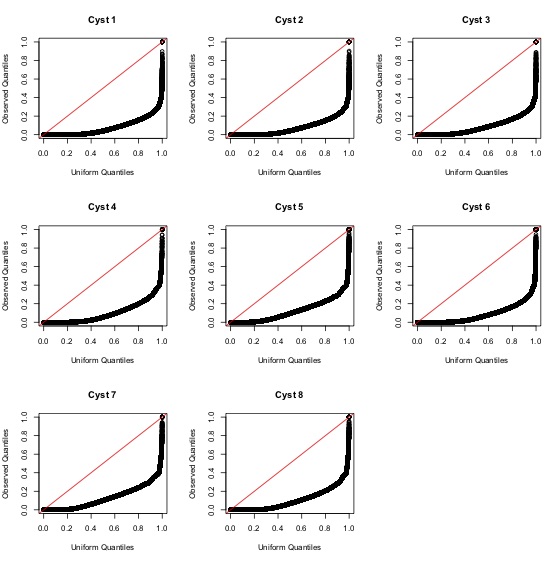
**

**Supplementary Figure S6: Distribution of *p* values in all analyses.** QQ-plots were generated for the ANOVA between non-ADPKD renal tissue and eight ADPKD cysts, the chi square analysis between all eight ADPKD cysts, and each Fisher’s Exact Test between the non-ADPKD reference methylome and a single ADPKD cyst. Generated in R using the lines of script: qqplot(qunif(ppoints(n)),p); abline(0,1,col=“red"), where n is number of tests and p is the list of associated *p* values. All observed quantiles lie below the quantiles of the uniform distribution as indicated by the 45-degree line.

**Supplementary Figure S7:** **Top 20 gene ontology terms enriched in the intragenic fragments associated with ICVs.** The ICVs associated with intragenic regions of the genome (*n* = 4,204) are enriched for embryonic and morphogenic pathways (Supplementary Table S10, Intergenic region-associated ICV gene ontology in Supplementary Table S11).

**
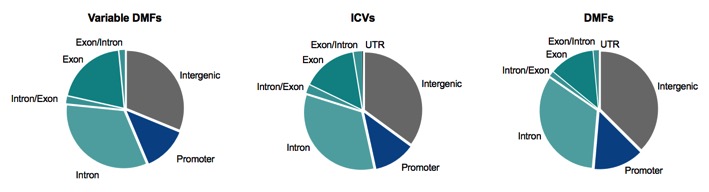
Supplementary Figure S8: Distribution of genomic features across DMFs and ICVs.** Variable DMFs (*n* = 837) are enriched for gene body-associated fragments compared to ICVs (*n* = 6,027) and DMFs (*n* = 2,024).
